# Seroprevalence of Foot-and-Mouth Disease in Susceptible Wildlife in Israel

**DOI:** 10.3389/fvets.2016.00032

**Published:** 2016-04-25

**Authors:** Ehud Elnekave, Roni King, Kees van Maanen, Hila Shilo, Boris Gelman, Nick Storm, Eyal Klement

**Affiliations:** ^1^The Robert H. Smith Faculty of Agriculture, Food and Environment, Koret School of Veterinary Medicine, The Hebrew University of Jerusalem, Rehovot, Israel; ^2^Israel Nature and Parks Authority (INPA), Jerusalem, Israel; ^3^The European Commission for the Control of Foot-and-Mouth Disease (EUFMD), Food and Agriculture Organization of the United Nations (FAO), Rome, Italy; ^4^Kimron Veterinary Institute, Beit Dagan, Israel

**Keywords:** FMD, wildlife, wild boar, NSP, prevalence

## Abstract

Foot-and-mouth disease (FMD) epidemics recur in Israel almost every year. Wild even-toed ungulates are seldom affected during these epidemics. The seroprevalence of FMD in wild ungulates during 2000 and 2005–2013 was estimated using anti-non-structural proteins ELISA. Overall, 209 samples were tested, comprising sera of 120 wild boar (*Sus scrofa lybicus*), 64 mountain gazelles (*Gazella gazella gazella*), 6 water buffaloes (*Bubalus bubalis*), and 19 Persian fallow deer (*Dama dama mesopotamica*). None of the tested animals presented clinical signs of FMD during blood collection. Sixteen samples [7.7% (95% confidence interval (CI_95%_) = 4.4–12.1%)] were found to be seropositive. Fifteen out of 120 samples (12.5%) from wild boar were seropositive, compared with only 1 out of 89 samples (1.1%) from all other species combined (Fisher’s exact test: *p* = 0.003). Most of the positive samples obtained from wild boar [13/15 (86.7%)] were collected during 2007, and analysis was restricted to that year and species only. The seroprevalence of FMD in this species during 2007 was estimated at 54.2% (CI_95%_ = 32.8–74.5%; *n* = 24). A significant infection cluster, comprising nine seropositive samples collected in three different locations, was identified in the north-eastern part of Israel. These findings indicate that wild boar was affected during the 2007 FMD epidemic, even though wild boar presenting FMD typical clinical signs were not observed during that year. The actual role of wild boar in the spread of FMD virus in this epidemic, however, could not be determined. The negligible seroprevalence of FMD found for all other surveillance years indicates that ongoing circulation of FMD among wildlife in Israel is unlikely. It is concluded that while the role of wildlife species in the dynamics of FMD in Israel is usually limited, there might be occasions, in which wildlife plays a part in the spread of the virus.

## Introduction

Foot-and-mouth disease (FMD) is a highly contagious viral disease, affecting cloven-hoofed ungulates (1) and causing major economic damage (2). Many wildlife species have been found to be susceptible to FMD infection, such as species of buffalo, deer, and wild boar (*Sus scrofa*) (3). Although wildlife species have been suggested as having contributed to FMD dynamics in several outbreaks (4, 5), their actual role in FMD dynamics was estimated to be of only limited significance (3, 6).

Foot-and-mouth disease epidemics have recurred, apart from in 2010, every year in Israel in the past decade. However, two out of 109 (1.8%) of the outbreaks that occurred during these epidemics affected wildlife: during 2007 in “Ramot Yissakhar” (mainly) in the Lower Galilee (north-eastern part of Israel); and next to the “Tzur Natan” settlement in the Sharon plain (the northern coastal plain of Israel). Both were caused by FMD virus of serotype O, affecting mountain gazelles (*Gazella g. gazella*) and resulting in severe clinical manifestations and even mortality (7, 8). A similar presentation, but with a higher percentage of mortality, was reported following the FMD outbreaks during 1985 among mountain gazelles in “Ramot Yissakhar” and the southern Golan Heights in the north of Israel (9).

Incursions of the FMD virus from surrounding countries into Israel have been previously demonstrated (10, 11). A possible role of wild ungulates in the spread of the disease was suggested, especially through the wild boar and mountain gazelles that are abundant in the northern part of Israel. Wild boar could also play a role in introducing the disease when crossing the borders with the surrounding countries. However, to date, the seroprevalence of FMD among wildlife species in Israel had never been estimated and published in the peer-reviewed literature. We have recently estimated the seroprevalence of FMD in small ruminants (12) and in cattle (Elnekave, personal communication) in Israel. The aim of this study was to expand the knowledge on FMD dynamics in Israel by (i) estimating the seroprevalence of FMD infection among wildlife in Israel and (ii) discussing its importance in the dynamics of FMD in Israel.

## Materials and Methods

### Study Population

Wild even-toed ungulate serum samples were collected by one of the authors (Roni King) during 2000 and 2005–2013. Overall, 244 samples were available, of which 35 samples were of poor quality for laboratory testing (i.e., hemolytic) and were therefore excluded. Consequently, 209 samples were tested, comprising 120 wild boar (*Sus scrofa lybicus*), 64 mountain gazelles, 6 water buffaloes (*Bubalus bubalis*), and 19 Persian fallow deer (*Dama dama mesopotamica*). The number of samples collected from each species and the year are provided in Table 1. Samples from mountain gazelle, Persian fallow deer, and water buffalo were collected either from injured wild animals or during immobilization performed to enable translocation of these animals. Samples from wild boar were mostly collected from hunted or severely injured wild animals that were euthanized.

**Table 1:** **Samples collected from wild ungulate species in Israel during 2000 and 2005–2013**.

Species	Collection years [# of samples (# of positive)]											
	2000	2005	2006	2007	2008	2009	2010	2011	2012	2013	Unknown	All
Wild boar (*Sus scrofa lybicus*)	1 (0)	–	1 (0)	24 (13)	7 (0)	46 (0)	8 (0)	9 (0)	15 (2)	8 (0)	1 (0)	120 (15)
Palestine mountain gazelle (*Gazella gazella gazella*)	–	4 (0)	5 (0)	8 (0)	11 (0)	6 (0)	12 (0)	4 (0)	7 (0)	7 (0)	–	64 (0)
Water buffalo (*Bubalus bubalis*)	–	–	–	4 (1)	–	–	–	2 (0)	–	–	–	6 (1)
Persian fallow deer (*Dama dama mesopotamica*)	–	–	–	2 (0)	3 (0)	6 (0)	6 (0)	1 (0)	1 (0)	–	–	19 (0)
Total	1 (0)	4 (0)	6 (0)	38 (14)	21 (0)	58 (0)	26 (0)	16 (0)	23 (2)	15 (0)	1 (0)	209 (16)

*The number of samples collected for each year and the number of positive samples, given in brackets, are indicated for each species*.

### Prevalence Estimation

Presence of antibodies specific to non-structural proteins (NSP) was detected using PrioCHECK^®^ FMD virus NS-blocking ELISA [Prionics Lelystad B.V., The Netherlands (currently owned by Thermo Fisher Scientific, Inc.)]. Tests were performed according to the manufacturer’s guidelines (http://www.fao.org/ag/againfo/commissions/docs/Workshop/nakuru_2010/PrioCHECK_FMDV-NS7610440_v1.2.pdf). The percentage of inhibition (PI) of each sample was calculated using the following formula:

$$\text{PI}=\left[100-\left(\frac{\text{OD}450\text{ test sample}}{\text{averaged OD}450\text{ of negative controls}}\right)\times 100\right]$$

In our study, serial testing, previously suggested by Paton et al. (13), was used in order to increase the test specificity. Seropositive samples (i.e., PI ≥ 50%) were therefore retested, and only samples found positive in two repeated tests were considered positive. FMD prevalence was thus calculated twice (i) using all positive samples found for the first test and (ii) using only positive results found for both tests.

In order to avoid over-estimation of FMD prevalence, we based the analysis only on the results that were positive in both tests.

### Data Analysis

Data obtained for the collected samples comprise the host species, sampling date, approximate location of sample collection, and also sex where possible. Although the age of the animals was not documented properly in all cases, the majority of samples were collected from animals older than 1 year, including all the samples that were eventually found to be seropositive. Data on FMD outbreak occurrence were obtained from the Israeli veterinary services (IVS) annual reports and from reports submitted to the OIE [based on the data published on the World Animal Health Information Database (WAHID)].

Using ArcGIS 10.0 (ESRI, Redlands, CA, USA), the samples’ approximate locations and the locations of outbreaks in both domestic species (during 2006–2007) and wildlife (in “Ramot Yissakhar,” see above) were mapped. Additionally, the Euclidean distances to the nearest FMD outbreak during 2006–2007 were calculated for wild boar samples collected during 2007. Disease clusters in wild boar collected during 2007 were identified using SatScan™ software (14).

Data were summarized using Microsoft Excel^®^ data spreadsheet. Data analysis was restricted to wild boar samples collected during 2007 (see below). The associations between the different variables and seropositivity were estimated. Fisher’s exact tests were performed to assess statistical significance of the association of seroprevalence with discrete variables, and a logistic regression model was fitted for continuous variables. Statistical analysis was performed using WinPEPI™ statistical package (15) and SPSS™ statistics version 21.0 (IBM Corp., Armonk, NY, USA). A significance level of α = 0.05 was applied.

## Results

**Figure 1:**
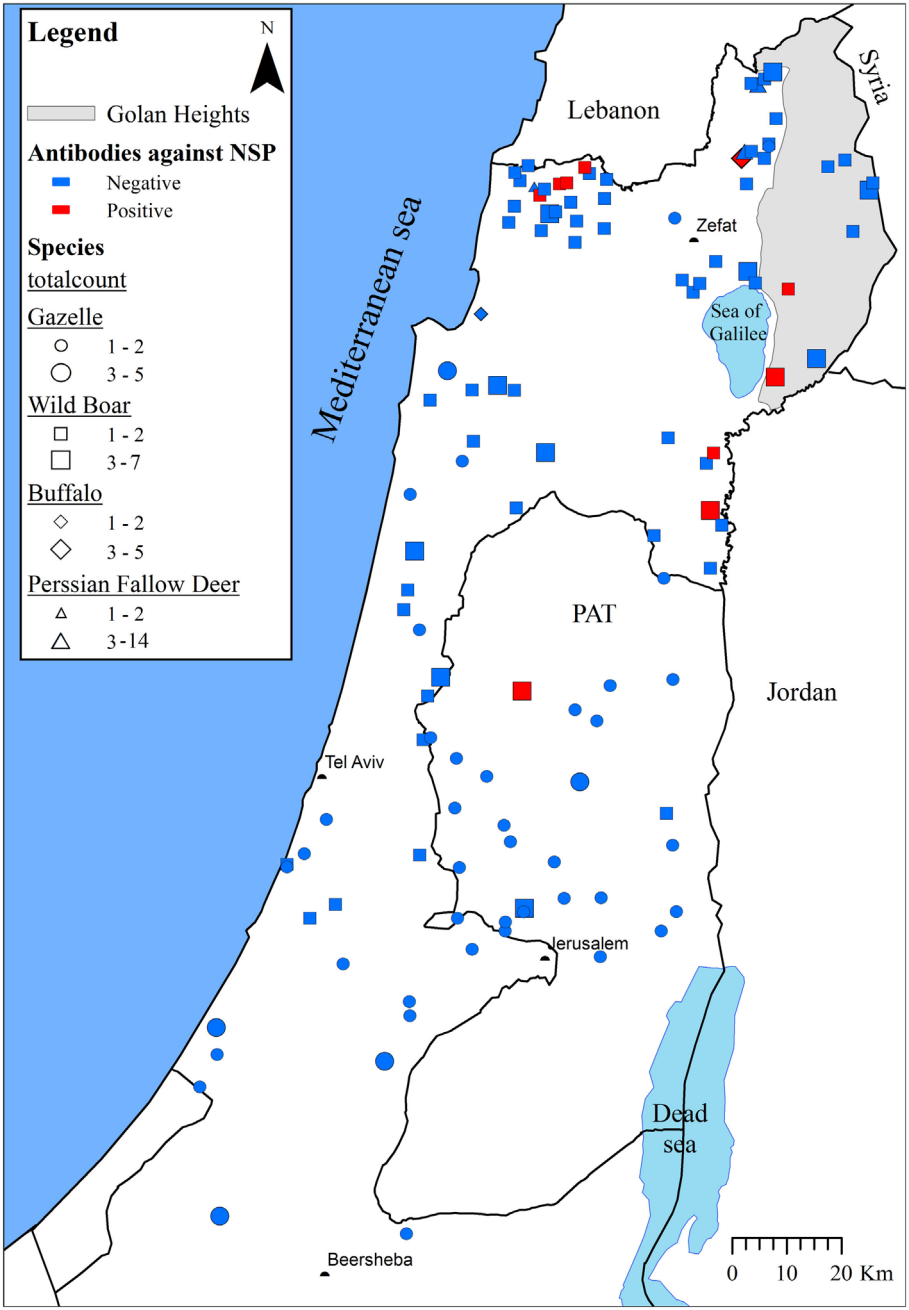
**The seroprevalence of foot-and-mouth disease virus in wildlife species in Israel during 2000 and 2005–13**. The approximate locations from which samples were collected are indicated. Wildlife species and the number of collected samples are illustrated by different shapes and sizes, respectively. A collection location was considered positive (marked red) if at least one of the species samples was found to be positive. Otherwise, the location was considered negative (marked blue).

None of the sampled animals presented clinical signs of FMD. A total of 17/209 animals [8.1% (95% confidence interval (CI_95%_) = 4.8–12.7%)] and 16/209 animals [7.7% (CI_95%_ = 4.4–12.1%)] were found to be seropositive using all positive results from the first test and only positive results on both tests, respectively. Most of the positive samples were collected in the northern part of Israel (Figure 1).

Fifteen out of 120 samples (12.5%) collected from wild boar were seropositive, compared to only one out of 89 samples (1.1%) obtained from all other species combined (Table 1; Fisher’s exact test: *p* = 0.003). Most of the positive samples obtained from wild boar [13/15 (86.7%)] were collected during 2007 (Table 1). Therefore, further analysis was restricted to wild boar samples collected during that year.

Thirteen out of 24 samples collected from wild boar during 2007 were positive (Table 1), and the FMD seroprevalence in wild boar during 2007 was estimated at 54.2% (CI_95%_ = 32.8–74.5%). A significant positive association was found between proximity to an outbreak and seropositivity (OR = 2.13, CI_95%_ = 1.06–4.27, *p*-value = 0.03, logistic regression).

Data on wild boar sex (female/male) were not available for 22 samples, and analysis of this variable was therefore based on a small data set. No significant association of sex with infection was found when only samples collected in 2007 were analyzed (*n* = 18; *p*-value = 0.304, Fisher’s exact test), or when samples collected from all years were analyzed (*n* = 98; *p*-value = 0.310, Fisher’s exact test).

**Figure 2:**
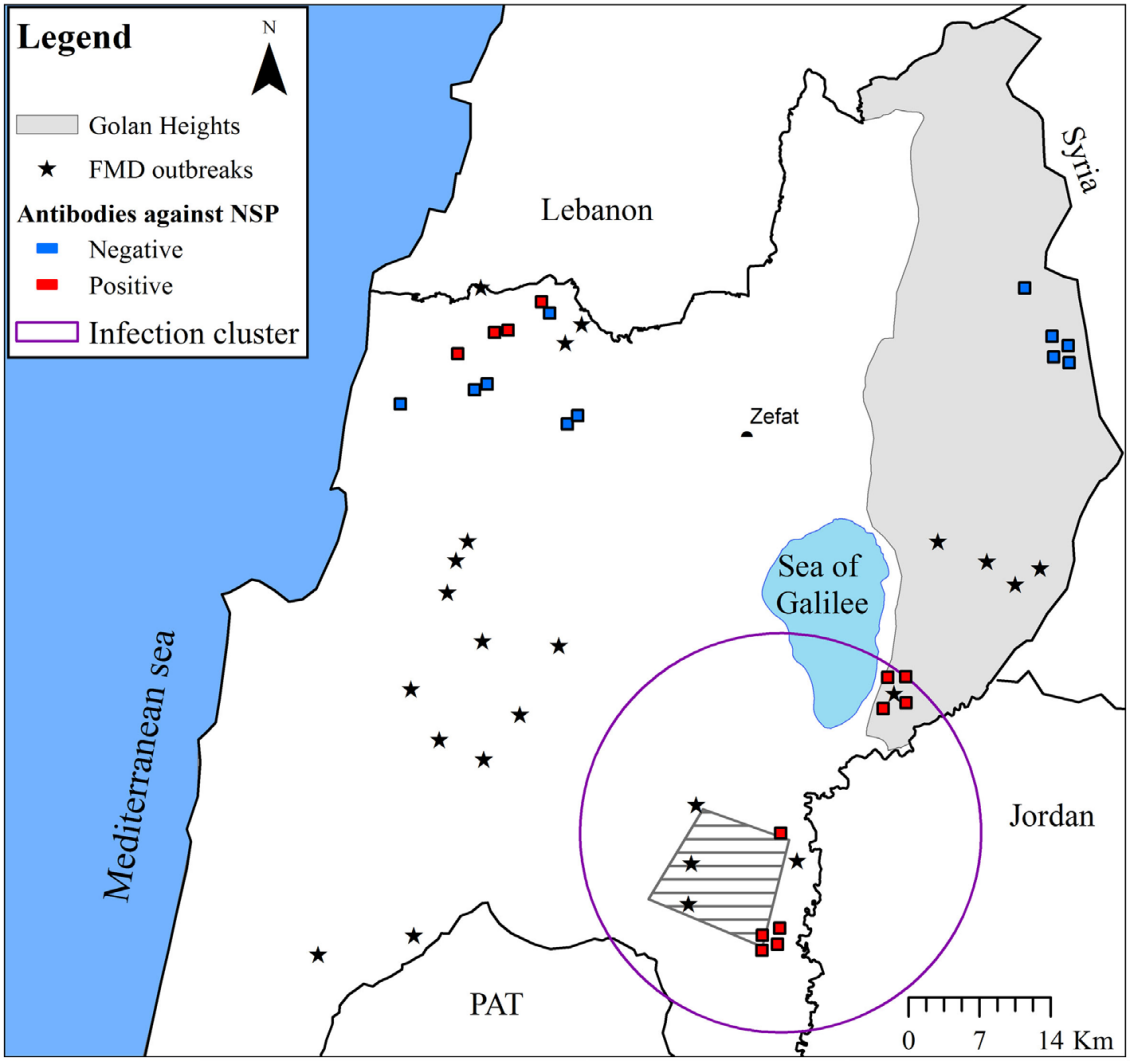
**The seroprevalence of FMD in wild boar in Israel during 2007**. The approximate locations from which samples were collected are indicated (samples collected from the same location were manually scattered around the location in order to allow better visualization). Positive samples are marked red and negative samples marked blue. FMD outbreak locations (during 2006–2007) are indicated by stars. Additionally, the approximate area of the main mountain gazelle population that was affected by FMD during 2007 (“Ramot Yissakhar”) is indicated by a gray polygon filled with diagonal lines. Significant prevalence cluster is indicated by a purple circle.

A significant infection cluster (coordinates: 32.612485 N, 35.535678 E; radius = 19.7km; and *p*-value = 0.002) was detected in wild boar samples collected during 2007. The cluster comprised nine seropositive samples from three different locations adjacent to FMD outbreaks (Figure 2).

## Discussion

The seroprevalence of FMD in different wildlife species in Israel sampled during 2000 and 2005–2013 is presented for the first time.

Fifty-seven percent and 31% of the samples were collected from wild boar and mountain gazelles, respectively. These two species are significantly more abundant in Israel than the Persian fallow deer and the water buffalo, which have been re-introduced into the wild in restricted locations in Israel. Thus, the present sampling provides a good representation of the wild even-toed ungulates that might play an important role in FMD dynamics in Israel.

Most of the seropositive samples were of wild boar collected during 2007. The seroprevalence in wild boar during this year was estimated at 54.2% (CI_95%_ = 32.8–74.5%). The infection cluster detected in the north-eastern part of Israel comprised nine positive samples collected from three locations, adjacent to the FMD outbreaks that occurred during 2007, and a positive association was found between the proximity to an FMD outbreak and seropositivity. A similar association between seropositive results and proximity to outbreak centers was reported in Bulgaria, following the FMD epidemic there in 2011 (16). However, a lower seroprevalence (of 6.9 and 11.5%) was estimated in wild boar in Bulgaria and the adjacent area in Turkey, respectively (16, 17). This might indicate of differences in the virus transmission to wildlife during those outbreaks (e.g., higher infectiousness). The high seroprevalence in wild boar in Israel indicates that these animals were probably infected during the 2007 FMD epidemic in Israel, even though none of the sampled animals presented clinical signs of FMD during sampling, and there was no other evidence (i.e., reports on lameness in wild boar or animals displaying poor body condition) that indicated clinical signs of FMD in wild boar during this epidemic. Additionally, seropositive samples were collected only from wild boar older than 1 year, making it possible that these animals had been infected before 2007 and remained seropositive due to the longevity of antibodies to NSP (18). However, this scenario is less likely, as FMD infection had not been detected at all in wildlife in the few years prior to 2007.

The transmission of FMD from wild to domestic even-toed ungulates has been suggested in several studies, such as in antelopes (impala or kudu) infecting cattle in Zimbabwe (5) and the FMD outbreak in Bulgaria, where the index case was a wild boar with clinical signs of FMD (4, 16). Additionally, experimental studies have demonstrated the transmission of several FMD serotypes from wild boar to other wild boar and to domestic pigs, despite the variable levels of clinical presentation in the infected wild boar (19, 20). These findings, combined with the high seroprevalence found in wild boar in Israel during 2007, especially in the north-eastern part of Israel, may suggest that wild boar could have played a role in the disease transmission during that year.

The almost complete absence of seropositive samples in all years, but 2007, indicates that ongoing circulation of FMD virus among wildlife species in Israel is unlikely. This is corroborated by the absence of clinical infections in wildlife in Israel throughout those years (based on the data published on the WAHID interface and in the IVS yearly reports). These results are in accordance with previous studies suggesting that, apart from the African buffalo (*Syncerus caffer*) that was found to be an infective carrier of FMD virus (6), other wildlife species are not capable of carrying the FMD virus for long periods (3, 6).

While the wild boar population in Israel continues to increase (21), the size of the two main mountain gazelle populations in Israel (i.e., in “Ramot Yissakhar” and southern Golan Heights) has significantly decreased since 1985, especially in the southern Golan Heights (22). This decrease, leading to lower densities of mountain gazelles, can partially explain the rare FMD occurrence in this wildlife species, while adjacent livestock populations are more frequently affected. Morgan et al. (23) demonstrated that small-size wildlife populations will fail to propagate an FMD epidemic. Several additional explanations may also be suggested, such as (i) variability in the virulence of different FMD serotype and subtypes can lead to higher infection and transmission rates of the wildlife species (3, 6); (ii) variability in the susceptibility of different wildlife species to infection (for most species of wild ungulates the susceptibility is unknown) (3, 6); and (iii) fluctuations in the wildlife population densities in certain locations throughout the year (e.g., as a result of food or water abundance) may influence the risk of disease transmission within the population and between wildlife and livestock (6).

## Conclusion

A negligible seroprevalence of FMD was found in the wildlife in Israel for all surveillance years but 2007. During 2007, wildlife species were clinically and subclinically affected by FMD. These findings indicate that an ongoing circulation of FMD among wildlife in Israel is unlikely, and that the wildlife species’ role in the dynamics of FMD in Israel is probably limited during most years. However, in certain years, infected wildlife species might play a role in contributing to the virus dynamics in Israel.

## Author Contributions

EE (equal contributor) – conception and design, analysis and interpretation of data, drafting of manuscript, critical revision, and statistical analysis. RK (equal contributor) – conception and design, acquisition of data, analysis and interpretation of data, and critical revision. KM – analysis and interpretation of data, and critical revision. HS, BG, and NS – analysis and interpretation of data. EK (advisor) – conception and design, analysis and interpretation of data, critical revision, and statistical analysis.

## Conflict of Interest Statement

The authors declare that the research was conducted in the absence of any commercial or financial relationships that could be construed as a potential conflict of interest.
